# Gene expression in cortex and hippocampus during acute pneumococcal meningitis

**DOI:** 10.1186/1741-7007-4-15

**Published:** 2006-06-02

**Authors:** Roney S Coimbra, Veronique Voisin, Antoine B de Saizieu, Raija LP Lindberg, Matthias Wittwer, David Leppert, Stephen L Leib

**Affiliations:** 1 Institute for Infectious Diseases, University of Bern, Friedbühlstrasse 51, CH-3010, Bern, Switzerland; 2 F. Hoffman-La Roche Ltd., Pharmaceutics, Basel, Grenzachertrasse 124, CH-4070, Basel, Switzerland; 3 Department of Research, University Hospitals Basel, Klingelbergstrasse 50, CH-4050, Basel, Switzerland

## Abstract

**Background:**

Pneumococcal meningitis is associated with high mortality (~30%) and morbidity. Up to 50% of survivors are affected by neurological sequelae due to a wide spectrum of brain injury mainly affecting the cortex and hippocampus. Despite this significant disease burden, the genetic program that regulates the host response leading to brain damage as a consequence of bacterial meningitis is largely unknown.

We used an infant rat model of pneumococcal meningitis to assess gene expression profiles in cortex and hippocampus at 22 and 44 hours after infection and in controls at 22 h after mock-infection with saline. To analyze the biological significance of the data generated by Affymetrix DNA microarrays, a bioinformatics pipeline was used combining (i) a literature-profiling algorithm to cluster genes based on the vocabulary of abstracts indexed in MEDLINE (NCBI) and (ii) the self-organizing map (SOM), a clustering technique based on covariance in gene expression kinetics.

**Results:**

Among 598 genes differentially regulated (change factor ≥ 1.5; p ≤ 0.05), 77% were automatically assigned to one of 11 functional groups with 94% accuracy. SOM disclosed six patterns of expression kinetics. Genes associated with growth control/neuroplasticity, signal transduction, cell death/survival, cytoskeleton, and immunity were generally upregulated. In contrast, genes related to neurotransmission and lipid metabolism were transiently downregulated on the whole. The majority of the genes associated with ionic homeostasis, neurotransmission, signal transduction and lipid metabolism were differentially regulated specifically in the hippocampus. Of the cell death/survival genes found to be continuously upregulated only in hippocampus, the majority are pro-apoptotic, while those continuously upregulated only in cortex are anti-apoptotic.

**Conclusion:**

Temporal and spatial analysis of gene expression in experimental pneumococcal meningitis identified potential targets for therapy.

## Background

Bacterial meningitis (BM) is associated with high mortality (~30%) and morbidity [1,2]. Up to 50% of BM survivors are affected by neurological sequelae that are due to a wide spectrum of brain injury including neuronal necrosis in the cortex (CX) and apoptotic neuronal death in the hippocampus (HC) [3-5]. Despite this significant disease burden, the genetic program that regulates the mechanisms leading to brain damage as a consequence of BM is largely unknown. High-throughput methods, e.g. DNA microarrays, can provide a comprehensive picture of the genes underlying the host responses to BM. This knowledge is a prerequisite for understanding the pathogenesis of brain damage and can drive the development of new therapeutic modalities for BM.

The evaluation of the functional significance of large groups of genes constitutes the real challenge for microarray users. Clustering genes according to their expression patterns may reveal only a partial picture of the biological implications of the data. To overcome this problem, methods that extract knowledge from the scientific literature by gene-name co-citation frequencies [6,7] or by recognizing patterns of word occurrences [8] have been used. More recently, a technique has been developed to cluster genes automatically on the basis of the frequencies of words present in abstracts indexed in the National Center for Biotechnology Information (NCBI) PubMed MEDLINE database [9].

The aim of this study was to identify genes and sets of genes implicated in the pathophysiological mechanisms leading to the neuronal damage observed in BM. We used an infant rat model of pneumococcal meningitis and DNA microarray technology to assess gene expression profiles in the brain regions known to be preferentially damaged, i.e. CX and HC in mock-infected controls and during the early (22 h) and late (44 h) phases of acute BM. To analyze the data, we implemented a bioinformatics pipeline for gene clustering combining literature profiling [9] and co-variance analysis of expression kinetics [9,10].

## Results

### Animal model

Eighteen hours after infection, all infected animals (n = 10) had meningitis as evidenced by the clinical status and positive bacterial titers in the cerebrospinal fluid (CSF) (log_10 _7.3 ± 0.6 cfu/ml).

### Microarrays

In total, 598 Affymetrix probe sets showing a change factor ≥ 1.5 (p ≤ 0.05) were selected. These represent 458 unique named genes and 67 expressed sequence tags (ESTs)/unnamed genes that were differentially regulated in the CX and/or in the HC when at least two of the defined conditions were compared (i.e.: CX 22 h vs. CX mock-infected, OR CX 44 h vs. CX mock-infected, OR CX 44 h vs. CX 22 h, OR HC 22 h vs. mock-infected, OR HC 44 h vs. mock-infected, OR HC 44 h vs HC 22 h).

### Literature profiling

**Figure 1:**
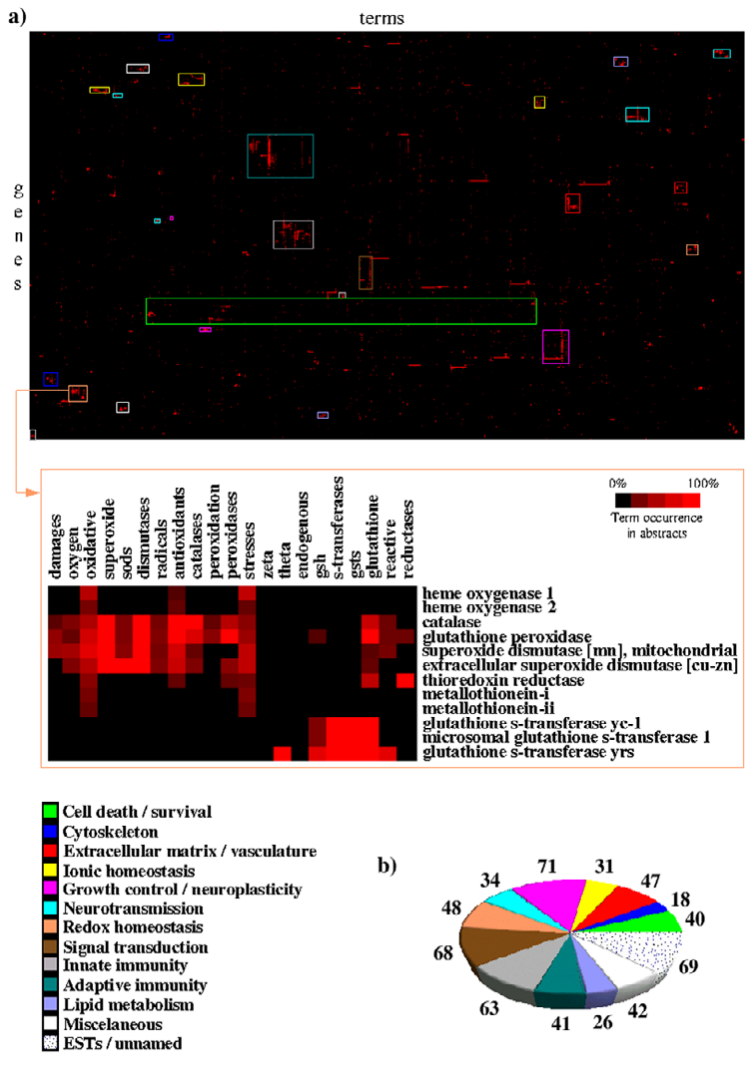
**Distribution of probe-sets in automatically disclosed functional groups by literature-profiling**. (a) Clustergram showing 444 named genes clustered according to the similarities between the patterns of occurrence of 1074 terms in their abstracts; detail showing one cluster of genes related to redox homeostasis. (b) The number of probe-sets in each functional group.

Between 1 and 100 abstracts were found in MEDLINE for 454 out of the 458 named genes represented in the GeneChip^® ^Rat Genome U34A by 598 probe-sets (total 32973 abstracts). The term-by-gene matrix generated comprised 444 genes and 1074 valid terms (ten genes had no characteristic term passing the filters). Twenty-eight clusters of genes forming nodes in the clustergram with a correlation varying from 0.114 to 0.948 were extracted and, in 25 cases, two or more clusters were manually merged to compose a larger group of genes with related functions. Eleven functional groups that may play a role in the host response to BM were identified. Genes not related to any pathophysiological mechanism known to be relevant in BM were categorized as "Miscellaneous". Seventy-seven per cent (462/598) of the probe sets were automatically assigned to one of the 11 functional groups (or to the ESTs/unnamed genes, or the miscellaneous groups) with 94% accuracy (manually checked). Twenty-nine genes were found to fit better in a functional group different from the one to which they were automatically assigned. The remaining 136 probe-sets that were not grouped by the automatic approach were manually transferred to one of the 13 groups (Figure 1).

### Self-organizing map

**Figure 2:**
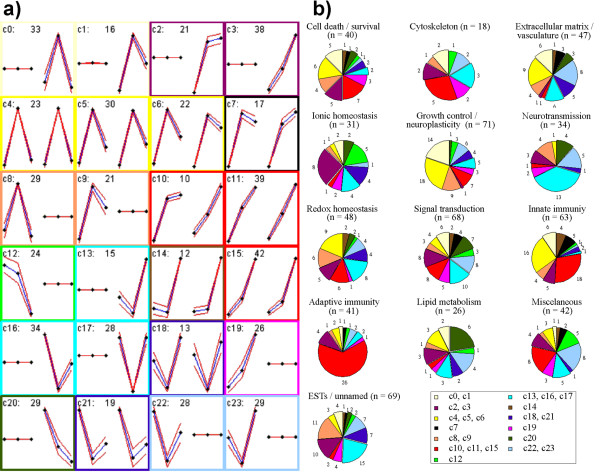
**Gene clustering by self-organizing maps (SOM) (a, b) and literature-profiling (b)**. (a) Genes clustered according to their expression dynamics (SOM): twenty-four clusters were disclosed by the SOMs algorithm, which were grouped into 6 patterns, i.e.: (1) genes transiently upregulated in the acute phase (yellowish); (2) genes continuously upregulated in the acute and the late phase (reddish); (3) genes transiently downregulated in the acute phase (bluish); (4) genes continuously downregulated in the acute and the late phase (greenish); (5) genes transiently upregulated in CX and continuously upregulated in HC (black); (6) genes transiently downregulated in CX and continuously upregulated in HC (brown). Patterns 1, 2 and 3 are subdivided into "mega-clusters" comprising clusters of genes differentially regulated only in CX, or only in HC, or in both tissues concomitantly. Pattern 4 contained two clusters, one with genes continuously downregulated only in CX, the other with genes continuously downregulated only in HC. In each cluster diagram, the three points on the left side represent the average gene expression levels in the CX in mock-infected animals, and in infected animals in the acute and the late phase, respectively. The three points on the right side refer to gene expression in the HC at these time points. c*n *is the identification of each cluster, and the following values correspond to the number of probe-sets comprising the cluster. Clusters c4, c5, c6, c7, c10, c11, c14, c15, c18 and c21 contain 227 probe-sets representing genes with a similar gene expression pattern in CX and HC; Clusters c8, c9, c12, c19, c22 and c23 contain 157 probe-sets representing genes differentially regulated only in the CX; Clusters c0, c1, c2, c3, c13, c16 and c17 contain 214 probe-sets representing genes differentially regulated only in the HC. (b) Combined clustering obtained with self-organizing maps and literature-profiling: Cold colors (bluish and greenish) represent genes that were downregulated, and warm colors (yellowish and reddish) represent genes that were upregulated. The corresponding clusters disclosed by the SOM algorithm are indicated in the box, following the same color schema of Figure 3(a). Detached pieces in the pies represent genes differentially regulated only in the HC.

The SOMs algorithm disclosed 24 clusters of probe-sets representing 6 expression kinetic patterns (Figure 2):

1) Transient upregulation in early acute meningitis (29.1 %);

2) Continuous upregulation in early and late acute meningitis (29.4 %);

3) Transient downregulation in early acute meningitis (27.8 %);

4) Continuous downregulation in early and late acute meningitis (8.9 %);

5) Transient upregulation in early acute meningitis in the CX, and continuous upregulation in early and late acute meningitis in the HC (2.8 %);

6) Transient downregulation in early acute meningitis in the CX and continuous upregulation in early and late acute meningitis in the HC (2.0 %).

### Overview of differential gene regulation

Overall, genes associated with growth control/neuroplasticity, signal transduction, cell death/survival, cytoskeleton, innate and adaptive immunity were upregulated. In contrast, most genes related to neurotransmission and lipid metabolism were downregulated. The majority of the genes associated with ionic homeostasis, neurotransmission, signal transduction and lipid metabolism were differentially regulated only in the HC. The other functional groups identified were: redox homeostasis and extracellular matrix/vasculature (Figures 1 and 2). Table 1 presents a list of 102 probe sets and their respective expression values in the CX and in the HC at the three time-points. They represent the 83 genes discussed below. These genes were selected because they were continuously up- or down-regulated during the early and late phases of acute BM, thus representing potential targets for therapeutic intervention at the time of onset of pathophysiological processes leading to brain injury. Eighty percent of these genes (66/83) have not previously been reported in BM.

## Discussion

### Functional analysis

**Figure 3:**
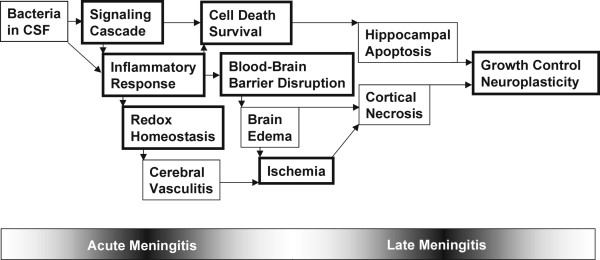
**Schematic representation of the pathophysiological events during acute and late meningitis**. Functional groups listed in Table 1 are marked with a bold lined box.

The pathogenesis of brain damage in BM arises from the interplay of bacteria and the host inflammatory response. It is generally accepted that the release of bacterial products (such as peptidoglycan and lipoteichoic acid, lipopolysaccharide, pneumolysin and bacterial DNA) into the CSF triggers the inflammatory response in the subarachnoid space by inducing the production and release of inflammatory cytokines, chemokines and lipid inflammatory mediators. These bacterial products also upregulate adhesion molecules in brain vascular endothelial cells and promote the recruitment of granulocytes into the CSF leading to the pronounced pleocytosis characteristic of BM. Granulocytic inflammation has a central role in the complex central nervous system (CNS) alterations associated with BM [11-14]. The pathophysiology of BM can be summarized as a sequence of sometimes overlapping processes that culminate in neuronal death of either the necrotic or apoptotic type in the CX or HC, respectively (Figure 3).

### Signaling cascade

It is difficult to assign biological functions to individual signal transduction molecules or transcription factors in the context of BM since they are likely to play a role in modulating different activities in many cell types within the infected brain. Our intention here is to highlight hypotheses arising from our results that are supported by literature data. Further investigations are required to test these hypotheses.

One of the first steps in the host immune response to BM is the activation of Toll like receptor 2 by binding of peptidoglycan (and/or lipoteichoic acid) to the CD14 monocyte membrane receptor (Table 1, #01, #02) [15]. Soluble CD14 (sCD14) can also act as an inflammatory co-ligand *in vivo*. In a murine model of pneumococcal meningitis increased CSF concentrations of sCD14 correlates with CD14 transcriptional upregulation mainly in intrathecal leukocytes [16]. In the infant rat model of pneumococcal meningitis, the two distinct expression kinetics of CD14 observed in the CX and in the HC suggest that parenchymal cells, most likely astrocytes and microglia, also contribute to the inflammatory cascade by increasing CD14 expression. This idea is further supported by our findings that CD14 and TNF-alpha (Table 1, #20) were both continuously upregulated only in the HC in the early and late phases of acute BM. TNF-alpha is known to activate the expression of CD14 [17].

Once triggered by the activation of Toll like receptor 2, the inflammatory cascade characteristic of BM is initiated by cytokines. The signalling events induced by cytokines include activation of an appropriate G coupled protein complex and stimulation of phospholipases the products of which activate a subset of protein kinase C leading to the phosphorylation of other signalling proteins.

Our data suggest a role for the G protein-coupled receptor VTR 15–20 (Table 1, #03) in the late events of the acute inflammatory reaction in the HC that leads to neuron death by apoptosis. The VTR 15–20 is well known to modulate neuro-immune function, and its expression in brain and spleen is regulated by immunological challenge [18].

The continuous upregulation of phospholipase A2 (Table 1, #06) from early to late acute BM might prolong the inflammation in the HC since this enzyme is required for the production of protaglandins, leukotrienes and platelet-activating factor by inflammatory cells in response to stimuli [19,20].

Phospholipase D1 (Table 1, #07) hydrolyzes phosphatidylcholine to generate phosphatidic acid, and choline, an important mechanism of cell signal transduction (reviewed in [21]). Our data suggest this to be mainly a component of the cortical signalling cascade in response to BM.

Activation of protein kinase C-delta (Table 1, #04, #05) is crucial for neutrophil apoptosis [22] ensuring the resolution of the inflammatory response. This is possibly part of an intrinsic mechanism to limit the extent of the inflammation in the CX in pneumococcal meningitis. Interestingly, we found no evidence of the activation of this mechanism in the HC.

Lipocortin III (Table 1, #95), or annexin III, an enzyme of inositol phosphate metabolism, can down-modulate the inflammation through inhibition of phospholipase A2 (see above) [23]. The simultaneous upregulation of lipocortin III and phospholipase A2 in the HC during the early and late acute BM suggests that these two molecules might interact in a feedback loop to modulate the local inflammatory response.

The phosphatidylinositol 3' kinase (PI3K, the p55 subunit of which was transiently downregulated only in the HC in the present study – data not shown) pathway plays a central role in regulating numerous biological processes known to be relevant to the pathophysiology of BM, including cell adhesion, migration, activation and survival [24,25]. However, the involvement of PI3K and its modulators in the pathophysiology of BM has not yet been proved. Another component of the inositol signalling system, inositol polyphosphate 5' phosphatase (SHIP) (Table 1, #10), can negatively modulate the PI3K signalling pathway by hydrolyzing the second messenger PI-3,4,5-trisphosphate generated by PI3K [24]. The upregulation of SHIP might inhibit the PI3K signalling pathway, ultimately triggering the apoptotic program in HC neurons.

It has recently been reported that inhibiting tyrphostin AG 126-sensitive tyrosine-protein kinase pathways improves the outcome in experimental pneumococcal meningitis [26]. According to our data, Lyn and p56-hck (Table 1, #11–#13) are the only tyrosine-protein kinases to be highly upregulated during the early and late phases of acute BM, making them potential targets for inhibition by tryphostin AG126. However, the sensitivity of Lyn and p56-hck to inhibition by AG126 has not been proven to date. Nevertheless, Lyn belongs to the Src tyrosine-protein kinase family that is involved in the CD36-dependent signalling cascade initiated by beta-amyloid in the Alzheimer's brain. Target disruption of Src kinases downstream of CD36 inhibits macrophage inflammatory responses to beta-amyloid, including production of reactive oxygen species (ROS) and chemokines, and results in decreased recruitment of microglia to sites of amyloid deposition *in vivo *[27].

Bruton's tyrosine kinase (Btk) (Table 1, #14) is a key regulator of LPS-induced TNF-alpha production. Over-expression of Btk results in stabilization of TNF-alpha mRNA [28]. Interestingly, Btk and TNF-alpha (Table 1, #20) presented opposite transcriptional kinetics in the HC, i.e. while TNF-alpha mRNA levels progressively increased in the course of the disease, Btk mRNA levels decreased.

In the hippocampus, CaM kinase-Gr (Table 1, #15) is localized to the processes and nuclei of developing neurons. This enzyme regulates developing neuron's sensitivity to Ca^2+ ^at different subcellular levels [29]. We have previously shown that pneumococcal meningitis triggers the apoptotic cell death cascade preferentially in progenitor cells and immature neurons in the dentate gyrus [30].

### The inflammatory response

Increased CSF concentrations of the proinflammatory cytokines TNF-alpha, IL-1-beta, IL-6, IL-8 and the anti-inflammatory IL-10 are characteristic for BM [2]. TNF-alpha, IL-1-beta and IL-6, the major early-response cytokines, trigger a cascade of inflammatory mediators including other cytokines, chemokines, arachidonic acid metabolites, reactive nitrogen and oxygen intermediates and proteases [5,31].

In the present study, IL-1-beta and IL-6 were transiently upregulated in the CX and in the HC during the early phase of acute BM (data not shown); no changes in the expression of IL-8 and IL-10 were observed at the time points investigated. Also, no changes in TNF-alpha (Table 1, #20) expression were observed in the CX. TNF-alpha stimulates the expression of chemokines and adhesion molecules, which facilitate the passage of leukocytes from the circulation into the subarachnoid space. In addition, TNF-alpha augments the expression of major histocompatibility complex molecules (Table 1, #36–#46) and thus facilitates the cytolytic action of T-lymphocytes [2]. These are typical events of early acute BM and may correlate with an increase in the CSF levels of TNF-alpha at the very beginning of the inflammatory reaction, peaking at 12 h after infection, i.e. before the first assessment in this study (22 h) [5]. In the HC, however, TNF-alpha mRNA levels increased continuously in the early and late phases of acute BM. This is the first report describing increased TNF-alpha transcriptional activity in the late phase of acute experimental BM. The role of TNF-alpha in the late phase of acute BM in the HC is unknown and experimental approaches to decipher it have yielded disparate results [5,32,33]. Neurons with typical apoptotic phenotype in the dentate gyrus are detectable from ~20 h after experimental infection with pneumococci; the maximal number of apoptotic neurons has been reported at 36 hours after infection [34].

Increased concentrations of interleukin-1 beta converting enzyme (caspase-1; Table 1, #72, #73) and interferon-gamma-inducing factor (IL-18; Table 1 #21, #22) have previously been described in BM [2,35]. Indeed, caspase-1 activates IL-18 [36].

Signal transducer and activator of transcription 1 (STAT1; Table 1, #23) is directly activated by ROS in the brain. It participates in the regulation of cytokine-signalling and cellular responses, particularly to interferon-gamma. In addition, STAT1 is activated and translocated within ischemic neurons and may contribute to brain injury by regulating transcription and phosphorylation of proteins related to apoptosis and cell death [37].

5-lipoxygenase activating protein (FLAP; Table 1, #79) functions as a facilitator of 5-lipoxygenase (5-LOX) activity. The enzyme 5-LOX catalyzes the production of leukotriene A4 from free arachidonic acid released from membrane phospholipid by phospholipase A2 (Table 1, #06) [38]. Besides its role in leukotriene metabolism, some evidence suggests that FLAP is also an inhibitor of apoptosis [39].

### Blood-brain barrier disruption

The permeability of the blood-brain barrier (BBB) increases in BM [2] compromising homeostasis in the neural microenvironment. High levels of alpha-2 macroglobulin in the CSF correlate with BBB damage associated with BM [40]. Although leakage from plasma into the CSF may be important, our results suggest that a local up-regulation of the alpha-2 macroglobulin gene (Table 1, #34, #35) in the brain parenchyma may also contribute to its increased levels found in the CSF in BM.

The local production of angiotensinogen (*Agt*, Table 1, #47) by astrocytes in the brain parenchyma is required for maintenance of the BBB [41]. In spite of the downregulation of angiotensin in the HC, its transcriptional rate did not change in the CX, where, owing to the larger volume, it might have more dramatic effects on the integrity of the BBB. Rupture of the BBB is associated with the separation of intercellular tight junctions by breakdown of occludin and reorganization of the actin cytoskeleton [42]. In response, the transcriptional rates of genes encoding cell junction or cytoskeleton proteins were changed predominantly towards upregulation (Table 1, #53–#56, #94).

Metalloproteinases (MMPs) are produced as part of the immune response to bacteria. In addition to their activity as modulators of inflammation, they also degrade extracellular matrix proteins, increasing the permeability of the BBB [43] in early BM [44]. We have previously documented the transcriptional upregulation of MMP-3 (Table 1, #95–#97), -9 and -14 in infant rat brain tissue at 22 h after experimental pneumococcal meningitis [5,45]. Moreover, higher CSF levels of MMP-9 and TIMP-1 are associated with poor outcome in children with BM [46]. In the present study, MMP-9 and -14 were transiently upregulated only in the early phase of acute BM (data not shown). Interestingly, this is the first report of the upregulation of MMP-3 and the metalloproteinase inhibitor TIMP-1 (Table 1, #98, #99) in the late phase of acute experimental BM. On the protein level, the time course of TIMP-1 concentration within the cortices of rats with pneumococcal meningitis was assessed in a recent study [14]. The concentration of TIMP-1 protein peaked at 24 hours after infection (6.8-fold vs. sham infection) and subsequently decreased at 36 hours after infection (4.7-fold vs. sham infection). These findings are in good agreement with the data presented herein, where we found a 4.4-fold increase of TIMP-1 mRNA expression at 22 hours after infection and a 3.7-fold increase at 44 hours after infection.  The contribution of MMPs to the pathophysiological events occurring in the late phase of acute BM is still largely unknown. Treatment with different MMP inhibitors led to a significant reduction of mortality and reduced the extent of cortical damage, but only one compound combining the properties of MMP and TACE (TNF-alpha converting enzyme) inhibitor prevented neurons from undergoing apoptosis in the HC and preserved learning performance in survivors of experimental BM [5]. In the light of the above-mentioned data it is conceivable that specific MMPs might be involved in the late pathophysiological events leading to apoptosis in the hippocampal neurons in BM. Thus, MMPs and TIMPs may represent candidate targets for pharmacological modulation aimed at improving the outcome of BM.

### Redox homeostasis

Reactive oxygen species (ROS) and nitric oxide (NO) have been implicated as key mediators in the pathophysiology of BM [47], contributing, among other effects, to disruption of the BBB [2].

The multi-subunit enzyme complex NADPH oxidase (Table 1, #57) catalyzes the reduction of O_2 _into the superoxide anion O_2_^•^- in phagocytic cells as part of the host defence against invading microorganisms. Superoxide generated by NADPH oxidase(s) has been shown to be important for establishing an adequate inflammatory response to pneumococcal CNS infection [48]. However, superoxide, as well as other ROS, can also cause damage to the brain by oxidizing nucleic acids, proteins and membrane lipids.

During BM, hypoxanthine accumulates as a consequence of ATP breakdown [49]. Xanthine oxireductase converts hypoxanthine to xanthine and then to urate. This enzyme may be converted from the xanthine dehydrogenase form (Table 1, #58) to the xanthine oxidase form. The later uses molecular oxygen as electron acceptor, thereby generating superoxide and other ROS [50]. In advanced BM, urate accumulates in the CSF and CX and the activity of xanthine oxireductase, mainly in its innocuous dehydrogenase form, increases [51]. The oxidative damage associated with BM is inhibited by treatment with antioxidants reducing cerebral ischemic damage and preventing cerebral blood flow reduction [47,52]. Among the group of endogenous antioxidant enzymes, which includes superoxide dismutase (SOD), catalase and glutathione peroxidase (Table 1, #62, #63), the last-named was the only one we found to be continuously upregulated in the early and late phases of acute BM. SOD mitochondrial precursor was transiently upregulated in early acute BM in the CX and HC; catalase and extracellular SOD (copper-zinc SOD) were transiently downregulated in the early acute BM only in the HC (data not shown). Glutathione peroxidase reduces lipid hydroperoxide substrates to the corresponding hydroxy fatty acid, and then is regenerated to its native form by reduced glutathione. Glutathione S-transferases (GSTs) represent a major group of detoxification enzymes, which includes the membrane-bound isoenzyme microsomal GST 1 (Table 1, #59, #60). GSTs detoxify some of the toxic carbonyl-, peroxide- and epoxide-containing metabolites produced within the cell by oxidative stress. GSTs are strongly induced by ROS [53]. The expression kinetics of the above-mentioned redox homeostasis-related genes indicate that SOD and catalase are early mediators of the antioxidant defence mechanisms while the glutathione system is activated mainly in the late phase of acute BM.

Besides its function in catalyzing the first and rate-limiting step in heme degradation, heme-oxygenase-1 (HO-1; Table 1, #59, #60) may also play a protective role against oxidant-mediated injury. HO-1 is highly induced by some key mediators of the host response to BM such as inflammatory cytokines and prostaglandins [54].

### Ischemia

In advanced BM, cerebral blood flow is reduced causing cerebral ischemic injury and neuronal death [55,56]. We found two main proteins that play a role in controlling blood flow to be differentially regulated in this study. Tropomyosin (Table 1, #52), regulates the contraction of vascular muscle cells [57]. Tropoelastin (Table 1, #48), induce an endothelium-dependent vasorelaxation mediated by the elastin/laminin receptor and by endothelial NO production [58]. Upregulation of tropomyosin in the CX and downregulation of tropoelastin in the HC may account for the loss of vascular autoregulation and reduction in cerebral blood flow and ischemia in the late phase of acute BM.

Upregulation of glial fibrillary acidic protein (GFAP; Table 1, #24) is a marker of astrocyte activation. One aspect of astrocyte activation may be neuroprotection against excitotoxicity by uptake of excess glutamate and conversion to glutamine via the enzyme glutamine synthase. Our results indicate that astrocytes are already highly activated in the early acute BM and their activation persists well into the late phase of acute BM.

CX3CR1 (Table 1, #25), a G-protein coupled chemokine receptor, is expressed in the activated microglia cells of ischemic brain. In ischemia, the neuronally expressed chemokine fractalkine may participate in the activation and chemoattraction of microglia into the injured area acting through CX3CR1 [59]. Allograft inflammatory factor-1 (AIF-1; Table 1, #26–#27) is a putative calcium binding peptide also associated with microglia activation in the brain [60]. Our data on the transcription kinetics of CX3CR1 and AIF-1 indicate that microglia activation might reach maximal levels during the late phase of acute BM.

Metabolites of the kynurenine pathway, the metabolic pathway leading from tryptophan to NAD, have been implicated in several neuropathological conditions such as epilepsy, neurodegenerative disorders, global ischemia and neuronal death in the course of acute or chronic inflammatory diseases. The increased transcription of kynurenine 3-hydroxylase (Table 1, #65) in early and late acute BM can lead to the accumulation in the CX and HC of the potentially neurotoxic compounds 3-OH-kynurenine and quinolinic acid (QUIN), which may cause neuronal death of either the excitotoxic or apoptotic type. 3-OH-kynurenine is readily oxidized and gives rise to highly reactive hydroxyl radicals, which are known mediators of cell death. QUIN is an agonist of a subset of N-methyl-D-aspartate (NMDA) glutamate receptors. In contrast, when kynurenine, the substrate of the enzyme kynurenine 3-hydroxylase is available, kynurenic acid (KYNA) concentrations in CSF and in brain extracellular spaces increase significantly [61-64]. KYNA is an antagonist of the NMDA receptor and acts neuroprotectively (reviewed in [65]). Accordingly, adjunctive KYNA reduces neuronal injury in the CX and in the HC of infant rats with group B streptococcal meningitis [66].

### Cell death/survival

In pneumococcal meningitis, apoptosis has been reported as a major mechanism of damage to the hippocampus leading to learning and memory impairments following the disease [2,31]. In the infant rat, neuronal apoptosis caused by experimental pneumococcal meningitis was caspase-3 dependent and localized to the granule cell layer of the hippocampal dentate gyrus corresponding to immature neurons and/or neuronal progenitor cells [30].

Some archetypal pro-apoptotic genes, such as those encoding caspase-1 [67] and peripheral-type benzodiazepine receptor [68], were upregulated in the CX and in the HC (Table 1, #72, #74). Importantly, most genes included in the cell death/survival group that were continuously upregulated in early and late phases of acute BM only in the HC, e.g. those encoding cyclophilin C [69], apoptosis-inducing factor (AIF) [70], h-rev107 [71] and metastasis suppressor homolog [72,73] (Table 1, #66–#69)], are pro-apoptotic, and those continuously upregulated only in the CX. e.g. *Tyms *(thymidilate synthase) [74] and *Tep1 *(telomerase protein component 1) (Table 1, #70, #71) [75] are anti-apoptotic.

In the course of BM, the immunoreactivity of the caspase-3 precursor protein in hippocampal homogenates is decreased, paralleled by an increasing signal for active caspase-3 from 18 h after infection on [34]. These findings suggest that caspase-3 activity is mainly regulated at a post-translational level. Accordingly, transcription of caspase-3 was not differentially regulated in the present study.

Galectins (Table 1, #75–#78) are a family of carbohydrate-binding proteins defined by affinity for beta-galactoside and sequence homology of the carbohydrate-binding motif. Among the members of this family, Galectin-3 is the only one known so far to inhibit apoptosis, while galectin-9 is pro-apoptotic. Galectin-3 is expressed in a variety of cell types including activated microglia, sub-populations of dorsal root ganglia neurons and Schwann cells after nerve injury (reviewed in [76]). Galectin-9 induces apoptosis in T cell lines and other types of cell lines via the Ca^2+^-calpain-caspase 1 pathway [77]. In human astrocytes, Galectin-9 expression is enhanced by Il-1-beta [78]. Should the cellular sources and targets of galectin-3 and -9 be identified in the BM brain, their role in limiting the inflammatory reaction and/or neuron death may be clarified.

### Growth control/neuroplasticity

The results from this study indicate that besides the mechanisms directly driving cell death in the brain during BM, such as the activation of pro-apoptotic genes, brain injury is further modulated by mechanisms controlling cell growth and neuroplasticity.

TGF-beta1 [79] and activin [80] (Table 1, #85, #86) are both strongly induced in the infant rat brain following hypoxia-ischemia. In an rabbit model of pneumococcal meningitis, activin level in the CSF rose 15-fold in 24 hours and correlated positively with CSF protein content, microglia activation and the number of apoptotic neurons in the dentate gyrus [81]. According to our data, TGF-beta1 is upregulated at the transcriptional level while activin is not. This could be due to the high stringency threshold we used to define differential gene regulation, or to the intrinsically low sensitivity of microarrays. Our results do not exclude the possibility that transcription of activin is upregulated in the very early disease, before 22 h. Post-transcriptional activation should also be considered.

Neurotrophin-3 (NT-3; Table 1, # 88) is involved in the survival of neurons and the modulation of the immune system (reviewed in [82]). The transcriptional downregulation of NT-3 in the late phase of acute BM may account for the neuron loss in our infant rat model of BM. However, our results contrast with a previous report of elevated CSF levels of NT-3 in patients with BM [83].

Granulins (Table 1, #89–#90), also called epithelins, are peptides with growth factor modulatory effects on a variety of cells. They are expressed in a number of epithelia and in specific neurons in the brain, including pyramidal cells of the HC and defined neurons in the CX. Progranulin plays important roles in immunological and neuronal function [84]. Progranulin activates the PI3K signalling cascade, among others, and increases expression of cyclins B and D (Table 1, #81, #82). Intact progranulin is anti-inflammatory through the inhibition of TNF, while the proteolytic peptides generated by elastase may stimulate the production of inflammatory cytokines such as IL-18 [2]. 42A (Table 1, #90) is an S100-like protein of which the mRNA is induced in PC12 cells by nerve growth factor. S100 beta proteins stimulate neurite extension and neuron survival [85].

Glycine is a major inhibitory transmitter in some regions of the brain, including the HC, and its accumulation in the brain and CSF has been reported in animal models and in patients with BM [86,87]. On the one hand, excess of glycine can be excitotoxic by acting as an agonist of NMDA receptors. On the other hand, activation of ionotropic glycine receptors increases chloride conductance, hyperpolarizes the membrane and reduces neuronal excitability (reviewed in [88]). The downregulation of the glycine receptor subunit alpha-1 in the HC (Table 1, #93) may result in exacerbation of excitotoxicity.

### Miscellaneous

This group included a variety of genes that could not be included in any of the functional groups disclosed by the literature profiling approach. Within this group, heat shock protein Hsp27, proteasome subunits beta types 8 and 9 and proteasome activator complex subunits 1 and 2 were continuously upregulated (Table 1 #96, #98–#102).

Heat shock proteins (HSP) are potent regulators of apoptosis [89]. Hsp27 is an ATP-independent chaperone that confers protection against apoptosis through various mechanisms, including direct interaction with cytochrome c after its release from mitochondria [90], or Akt activation [91]. Hsp27 also enhances the degradation of ubiquitinated proteins by the 26S proteasome in response to stress-inducing stimuli such as TNF-alpha [92]. The ubiquitin-proteasome pathway is involved in the activation of NF-κB by enhancing degradation of its main inhibitor I-kappaBalpha. This function of Hsp27 would account for its antiapoptotic properties through the enhancement of NF-κB activity [93].

## Conclusion

High throughput analysis of gene expression in the CX and in the HC during the early and late phases of acute pneumococcal meningitis revealed functional groups of differentially regulated genes. The rat U34A chip contains only a subset of the entire rat genome, in comparison to current U230 versions that contain essentially whole genomes. Thus, the array used herein covers merely one third of the putative 30000 genes of the rat genome and therefore only a fragmented description of the rat transcriptome is at hand. The probesets on U34A represent the most highly annotated and well characterized genes known at the time it was produced and the selection may be biased towards genes that are already known to be important in critical pathophysiological processes. Nevertheless the selection of genes represented on the rat U34A chip is not biased towards a specific process and covers a wide range of the cellular transcriptional network. Our results are validated by the finding that most genes previously reported in BM are differentially regulated herein. Furthermore, evidence was obtained from the literature analysis to implicate a number of genes not previously known to play a role in the pathophysiology of BM. These genes represent 80% (62/82) of all those that were differentially regulated continuously in early and late phases of acute BM. In general, factors capable of promoting inflammation are continuously upregulated in the HC, but not in the CX, from early to late acute BM. This confluence of pro-inflammatory stimuli may account for a stronger and longer local response in the HC than in the CX; this phenomenon may favor apoptosis rather than necrosis as the predominant mechanism of cell death in hippocampal neurons. The data presented herein may provide a road map for further investigations into the pathophysiology of pneumococcal meningitis and may help to identify potential targets for adjuvant therapy of this disease.

## Methods

### Model of meningitis

An established infant rat model of pneumococcal meningitis was used as described previously [5]. The animal studies were approved by the Animal Care and Experimentation Committee of the Canton of Bern, Switzerland, and followed National Institutes of Health guidelines for the performance of animal experiments. Briefly, nursing Sprague-Dawley rats with their dams were purchased (RCC Biotechnology & Animal Breeding, Füllinsdorf, Switzerland), and infected (n = 10) on postnatal day 11 by intracisternal injection with 10 μl of saline containing log_10 _6.4 cfu/ml of *Streptococcus pneumoniae *(serogroup 3). The infecting organism was initially isolated from a patient with pneumococcal meningitis and has undergone multiple passages through infant rats in the course of experimental studies [14,94]. Mock-infected control animals (n = 5) were injected with 10 μl of saline. Eighteen hours after infection, the animals were weighed and the severity of the disease was scored using the following scale: 1 = coma; 2 = does not stand upright; 3 = stands upright within 30 seconds; 4 = minimal ambulatory activity, stands upright in less than 5 seconds; and 5 = normal [94]. Cerebrospinal fluid (CSF) was obtained by puncture of the cisterna magna and used for quantitative bacterial titers. Antibiotic treatment with ceftriaxone (100 mg/kg, subcutaneously q12 h; Roche Pharma, Reinach, Switzerland) was started. Animals were sacrificed with an overdose of pentobarbital (100 mg/kg, intraperitoneally) at 22 h for the mock-infected control group (n = 5) and for the group representing the early phase of acute BM (n = 5). Animals representing the late phase of acute BM (n = 5) were sacrificed at 44 h after infection.

### Tissue processing

Animals were perfused via the left cardiac ventricle with 30 ml of ice-cold, RNase-free phosphate buffered saline (PBS) followed by 30 ml of 50% RNAlater^® ^(Ambion Europe Ltd., Huntingdon, UK) in ice-cold, RNase-free PBS. Immediately afterwards, the meninges were removed from the brains. The hippocampi and the cortical hemispheres were dissected and stored separately in 150 μl of RNAlater^® ^at 4°C until isolation of RNA [5].

### RNA processing and hybridization

Tissue samples from CX and HC of each animal were processed and analyzed separately. Total RNA was extracted from brain samples using RNAzol (Tel-Test Inc., Friendswood, TX) and a commercially available kit (Bio 101, Carlsbad, CA) [5]. Total RNA was purified with RNeasy columns (Qiagen, Basel, Switzerland) before quantification and assessment of ribosomal RNA integrity on agarose gels. Double-stranded cDNAs were synthesized from 20 μg of total RNA using an oligo dT-T7 promoter primer (Roche Molecular Biochemicals, Mannheim, Germany). The cDNAs obtained were used as templates for *in vitro *transcription using the Megascript kit purchased from Ambion (Austin, TX) and biotinylated nucleotides (Bio-11-CTP and Bio-16-UTP) provided by Roche Molecular Biochemicals. Fragmented *in vitro *transcripts (cRNAs) were hybridized overnight on to commercially available rat microarrays containing 8799 rat specific probe sets (GeneChip^® ^Rat Genome U34A, Affymetrix, Santa Clara, CA). The hybridized samples were stained with streptavidin-R phycoerythrin (SAPE, Molecular Probes Inc., Eugene, OR) and the signal amplified using a biotinylated goat anti-streptavidin antibody (Vector Laboratories, Burlingame, CA) followed by a final staining with SAPE. Washing, staining and amplification were carried out in a fluidics station provided by Affymetrix. Microarrays were scanned in an Affymetrix GeneArray scanner (gain setting: 18,000). The image files obtained were analyzed using Microarray Suite 3.0 software (Affymetrix). The distribution of the samples on the microarrays was n = 4 for cortex and n = 5 for hippocampus in the mock-infected control group (n = 9); n = 5 for cortex and n = 5 for hippocampus in the group that was sacrificed at 22 hours after infection, in the early phase of acute BM (n = 10); and n = 5 for cortex and n = 5 for hippocampus in the animals that were sacrificed at 44 h after infection (n = 10), in the late phase of acute BM. Samples were not pooled and explants from CX and HC of each animal (n = 15) were processed and hybridized separately yielding a total of 29 independent readings.

### Raw data analysis

The Affymetrix^® ^.CEL files containing the unprocessed raw data of each rgu34a Array used in this study can be downloaded from [95].

Raw data analyses were carried out using RACE-A version2 software (F. Hoffmann – La Roche, Basel, Switzerland) [96]. For quantification of relative transcript abundance, the average difference value (Avg Diff) was used. All chips were normalized against the mean of the total sums of Avg Diff values across all 29 chips. We selected for further analysis only those probe sets with a reproducible change factor of their Avg Diff ≥ 1.5 (p ≤ 0.05) in the CX and/or HC when at least two of the three defined conditions were compared (i.e. early and late phases of acute BM, and mock-infected controls).

### Literature profiling

We implemented the literature-profiling algorithm of Chaussabel and Sher [9] in a Perl program (see Additional file 1: Our implementation of the literature profiling algorithm of Chaussabel and Sher). The output of our program can be described as a term-by-gene matrix of term-frequencies. The matrix was used to group genes by hierarchical clustering based on their shared vocabulary using the software packages Cluster and Tree View [97]. We applied the average linkage clustering algorithm to the two axes of the matrix (genes and terms) and the similarity metric method was the centered correlation. The results are visualized as a clustergram representing genes clustered according to their patterns of term occurrences (Figure 1).

### Self-organizing map (SOM)

SOM as implemented in the software GENECLUSTER 1.0 (MIT, Cambridge, MA) was used to group the 598 Affymetrix probe sets into clusters on the basis of similar expression kinetics over the three defined conditions for the CX and the HC independently. Avg diffs were converted to "zero" when no significant change was observed according to the criteria defined above in the section "Raw data analysis". The SOM algorithm reduces the dimensions of data through the use of self-organizing neural networks. GENECLUSTER 1.0 reduces dimensions by producing a bi-dimensional map that plots similar data items grouped together [10].

## Authors' contributions

RSC carried out analysis and interpretation of data, and drafted the manuscript.

VV and ABS carried out RNA processing and hybridization.

RLL participated in data analysis.

MW participated in revising the manuscript and preparation of data for download.

DL and SLL conceived and designed the study.

SLL coordinated the study and the animal experiments and participated in data interpretation and writing of  the manuscript.

## Supplementary Material

Additional file 1 Implementation of the literature profiling algorithm by Chaussabel and Sher. Click here for file

Additional file 2 Schematic of spatial overlap in gene expression between cortex and hippocampus. Click here for file

## Figures and Tables

**Table 1:** Probe-sets representing 83 genes, their expression levels in the CX and in the HC at three time-points, organized according to their functional groups and SOMs. In bold are genes previously known to play a role in the pathophysiology of BM. (-) = Avg Diff change factor ≥ 1.5, and/or p > 0.05 for all pairwise comparisons.

#	AFFY ID	REFSEQ ID	GENE	DESCR	CX mock-infected	CX early acute phase	CX Late acute phase	HC mock-infected	HC early acute phase	HC late acute phase	SOM
**Signal transduction**											

**01**	**AF087943_s_at**	**NM_021744.1**	***Cd14***	**monocyte differentiation antigen cd14 precursor **	**73.6**	**869.4**	**610.7**	**33.6**	**805.4**	**855.8**	**c7**
**02**	**AF087944mRNA_s_at**	**NM_021744.1**	***Cd14***	**monocyte differentiation antigen cd14 precursor **	**-**	**-**	**-**	**102.7**	**298.7**	**329.1**	**c2**
03	U76206_at	NM_133577.1	*Gpr105*	probable g protein-coupled receptor (vtr 15–20)	-	-	-	79.0	132.6	199.4	c3
04	M18330_at	NM_133307.1	*Prkcd*	protein kinase c, delta type (npkc-delta)	68.2	135.8	259.9	-	-	-	c19
05	M18330_g_at	NM_133307.1	*Prkcd*	protein kinase c, delta type (npkc-delta)	137.1	224.4	445.2	-	-	-	c19
06	U38376_s_at	NM_133551.1	*Pla2g4a*	phospholipase a2	-	-	-	341.1	637.3	992.7	c3
07	AB000778_s_at	NM_030992.1	*Pld1*	phospholipase d1	79.9	86.6	222.3	0.0	0.0	0.0	c19
08	U38812_s_at	XM_216233.1	*Itpr1*	inositol 1,4,5-triphosphate receptor type 1	5.03	14.47	125.6	-	-	-	c19
09	rc_AA859981_at	NM_172224.1	*Impa2*	myo-inositol monophosphatase 2	13.47	27.81	90.51	-	-	-	c19
10	U55192_at	NM_019311.1	*Inpp5d*	inositol ployphosphate 5' phosphatase (ship)	201.68	155.16	430.00	98.29	84.22	358.8	c14
11	rc_AA946044_s_at	NM_030857.1	*Lyn*	lyn tyrosine-protein kinase	34.15	84.15	172.62	22.02	68.72	192.35	c15
12	L14782_s_at	NM_030857.1	*Lyn*	lyn tyrosine-protein kinase	40.01	124.89	240.08	52.11	102.81	389.1	c15
13	S74141_s_at	NM_013185.1	*Hck*	tyrosine-protein kinase hck (p56-hck)	149.61	316.45	483.81	-22.42	409.21	690.65	c11
14	rc_AA892404_at		*Btk*	bruton's tyrosine kinase (btk)	-	-	-	134.22	46.21	-33.89	c20
15	M74488cds_s_at	NM_012727.1	*Camk4*	calcium/calmodulin-dependent protein kinase type iv catalytic chain (CaM kinase-Gr)	-	-	-	85.79	48.6	33.04	c20
16	U77038_g_at	NM_053908.1	*Ptph6*	protein-tyrosine phosphatase 1c (ptp-1c)	159.7	304.7	851.4	73.8	224.9	901.7	c15
17	U77038_at	NM_053908.1	*Ptph6*	protein-tyrosine phosphatase 1c (ptp-1c)	-55.5	16.4	165.0	-4.6	8.2	226.5	c15
18	L19180_at	NM_019140.1	*Ptprd*	receptor-linked protein tyrosine phosphatase(ptp-p1)	-	-	-	1106.6	524.8	243.7	c20
19	M10072mRNA_s_at	NM_138507.1	*Ptprc*	leukocyte common antigen variant 4 (cd45)	-22.25	28.57	132.68	-17.53	14.35	167.07	c15

**Innate immunity**											

**20**	**L00981mRNA#2_at**	**NM_012675.1**	***Tnf***	**tumor necrosis factor precursor (tnf-alpha)**	**-**	**-**	**-**	**-64.6**	**87.3**	**93.3**	**c2**
**21**	**AJ222813_s_at**	**NM_019165.1**	***Il18***	**interleukin-18 precursor**	**30.8**	**133.6**	**207.2**	**21.1**	**80.1**	**289.7**	**c11**
**22**	**U77777_s_at**	**NM_019165.1**	***Il18***	**interleukin-18 precursor**	**67.3**	**148.4**	**292.6**	**38.9**	**119.0**	**321.0**	**c15**
23	rc_AA892553_at	NM_032612.1	*Stat1*	signal transducer and activator of transcription 1	-90.9	295.2	654.8	-66.5	234.4	676.0	c11
**24**	**AF028784cds#1_s_at**	**NM_017009.1**	***Gfap***	**glial fibrillary acidic protein**	**534.9**	**3993.1**	**3184.2**	**923.9**	**4524.2**	**4733.2**	**c7**
25	U04808_at	NM_133534.1	*Rbs11*	cx3c chemokine receptor 1 (CX3CR1)	74.79	61.15	164.92	58.37	39.06	108.2	c14
26	U10894_s_at	NM_017196.1	*Aif1*	alograft inflammatory factor-1 (aif-1)	174.02	223.69	781.79	168.54	210.13	784.71	c14
27	U17919_s_at	NM_017196.1	*Aif1*	alograft inflammatory factor-1 (aif-1)	687.06	805.9	2154.2	620.75	733.07	2182.67	c14
28	X71127_at	NM_019262.1	*C1qb*	complement c1q subcomponent, b chain precursor	1158.3	1939.1	4410.7	981.7	1652.3	4279.6	c15
29	X71127_g_at	NM_019262.1	*C1qb*	complement c1q subcomponent, b chain precursor	134.4	507.1	1025.3	176.6	418.8	975.5	c15
**30**	**X52477_at**	**NM_016994.1**		**complement c3 precursor**	**-93.1**	**493.2**	**751**	**81.4**	**367.5**	**568**	**c11**
**31**	**M29866_g_at**	**NM_016994.1**		**complement c3 precursor**	**-76.3**	**465.2**	**953.3**	**20.8**	**439.3**	**993.2**	**c11**
**32**	**U42719_at**	**XM_215318.1**		**c4 complement protein mRNA**	**8**	**120.2**	**255.7**	**63.7**	**86.7**	**320.2**	**c15**
**33**	**rc_AI639117_s_at**	**XM_215326.1**		**alternative-complement pathway C3/C5 convertase**	**-519.8**	**818.2**	**1142.1**	**-382.7**	**1188.8**	**4030.51**	**c10**
**34**	**rc_AA900582_at**	**NM_012488.1**	***A2m***	**alpha-2-macroglobulin precursor**	**-**	**-**	**-**	**285.4**	**599.5**	**846.0**	**c3**
**35**	**M23566exon_s_at**	**NM_012488.1**	***A2m***	**alpha-2-macroglobulin precursor**	**-1469.4**	**-5.3**	**527.7**	**-609.4**	**307.7**	**2352.1**	**c11**

**Adaptive immunity**											

36	M31038_at	NM_012645.1	*RT1Aw2*	class i histocompatibility antigen, non-rt1.a alpha-1 chain precursor	-103.5	24.5	114.8	-	-	-	c19
37	M24324_f_at	NM_012645.1	*RT1Aw2*	class i histocompatibility antigen, non-rt1.a alpha-1 chain precursor	767.5	2015.0	3287.2	809.0	2211.3	4525.7	c11
38	M31018_f_at	XM_228021.1		rt1 class i histocompatibility antigen, aa alpha chain precursor	401.5	2335.9	5023.4	416.4	2893.1	7012.5	c15
39	M64795_f_at	XM_228021.1		h-2 class i histocompatibility antigen, d-37 alpha chain precursor	-186.5	-8.3	345.6	-133.0	-15.2	584.4	c15
40	L40362_f_at	XM_227984.1		h-2 class i histocompatibility antigen, d-k alpha chain precursor	189.5	1022.8	2404.2	124.3	1025.7	3378.6	c15
41	X13044_g_at	NM_013069.1	*Cd74*	h-2 class ii histocompatibility antigen, gamma chain	-58.7	273.0	2479.2	-79.8	335.3	2427.6	c15
42	X13044_at	NM_013069.1	*Cd74*	h-2 class ii histocompatibility antigen, gamma chain	3.5	219.0	1232.5	2.2	187.1	1139.5	c15
43	X14254cds_at	NM_013069.1	*Cd74*	h-2 class ii histocompatibility antigen, gamma chain	-68.3	14.9	238.6	-39.3	-10.1	230.2	c15
44	X14254cds_g_at	NM_013069.1	*Cd74*	h-2 class ii histocompatibility antigen, gamma chain	-247.3	-84.2	643.7	-200.6	-108.2	572.6	c15
45	X56596_at	XM_228006.1	*RT1.B-BETA(1)*	rt1 class ii histocompatibility antigen, b-1 beta chain precursor (rt1.b-beta(1)).	49.9	141.0	400.2	39.7	138.8	408.3	c15
46	X53054_at	XM_215333.1		rt1 class ii histocompatibility antigen, d-1 beta chain precursor.	-54.0	-7.4	77.3	-24.2	-15.9	96.1	c15

**Extracellular matrix/vasculature**											

47	M12112mRNA#3_s_at	NM_007428.2	*Agt*	angiotensinogen	-	-	-	227.7	75.3	87.6	c20
48	J04035_at	XM_237869.1	*Trela*	elastin precursor (tropoelastin)	-	-	-	254.9	51.9	-64.5	c20
**49**	**X02601_at**	**NM_133523.1**	***Mmp3***	**stromelysin-1 precursor (MMP3)**	**-**	**-**	**-**	**30.5**	**76.1**	**67.1**	**c2**
**50**	**rc_AI169327_at**	**NM_053819.1**	***Timp1***	**metalloproteinase inhibitor 1 precursor (TIMP1)**	**352.2**	**1573.0**	**1312.1**	**357.2**	**1475.4**	**1913.9**	**c7**
**51**	**rc_AI169327_g_at**	**NM_053819.1**	***Timp1***	**metalloproteinase inhibitor 1 precursor (TIMP1)**	**9.8**	**2099.0**	**1779.3**	**50.6**	**2171.3**	**3101.5**	**c7**

**Cytoskeleton**											

52	rc_AA859305_s_at	NM_173111.1		tropomyosin 3	60.7	143.4	184.4	-	-	-	c19
53	X62952_at	NM_031140.1	*Vim*	vimentin	1415.3	3269.2	4141.0	1032.8	3165.7	6517.2	c11
54	rc_AA892333_at	XM_147357.1	*Tuba6*	tubulin alpha-6 chain.	-58.1	1246.0	1762.2	34.0	989.2	2595.5	c11
55	X73524_at	NM_022531.1	*Des*	desmin	113.1	108.6	32.2	-	-	-	c12
56	rc_AA852046_s_at	XM_238547.1		gamma-smooth muscle isoactin promoter and exon 1	7.6	65.22	82.99	-	-	-	c19

**Redox homeostasis**											

**57**	**U18729_at**	**NM_024160.1**	***Cyba***	**nadph oxidase light chain subunit p22phox**	**-40.6**	**20.3**	**202.5**	**-109.7**	**5.4**	**255.8**	**c15**
**58**	**rc_AI172247_at**	**NM_017154.1**	***Xdh***	**xanthine dehydrogenase**	**-33.5**	**248.0**	**420.6**	**9.1**	**281.3**	**529.0**	**c11**
59	J02722cds_at	NM_012580.1	*Hmox1*	heme oxygenase 1	-3.4	258.0	389.9	-32.4	385.8	1074.1	c11
60	rc_AI179610_at	NM_010442.1	*Hmox1*	heme oxygenase	94.5	733.2	797.4	96.5	778.6	1995.6	c10
61	S81433_at	S81433.1		heme oxygenase-2	-154.9	-95.2	187.4	-	-	-	c19
62	X07365_s_at	NM_030826.2	*Gpx1*	glutathione peroxidase 1	1243.0	1709.8	3171.5	1286.8	1683.8	4025.0	c15
63	X12367cds_s_at	NM_030826.2	*Gpx1*	glutathione peroxidase 1	310.7	359.2	814.5	278.8	314.5	924.1	c14
64	J03752_at	NM_134349.1	*Mgst1*	microsomal glutathione s-transferase 1	40.8	36.5	110.9	43.5	39.2	428.1	c14
65	AF056031_at	NM_021593.1	*Kmo*	kynurenine 3-hydroxylase	58.4	80.5	153.2	56.2	64.2	209.3	c15

**Cell death/survival**											

66	C07012_f_at	NM_139096.1	*Ppicap*	cyclophilin c	-	-	-	283.5	750.3	1145.2	c3
**67**	**rc_AA891591_at**	**NM_031356.1**	***Pdcd8***	**apoptosis-inducing factor (Aif)**	**-**	**-**	**-**	**49.8**	**96.7**	**132.5**	**c3**
68	X76453_at	NM_017060.1	*Hrasls3*	h-rev 107	-	-	-	120.1	213.4	405.7	c3
69	rc_AI231213_g_at	XM_237691.1		metastasis suppressor homolog (Kai1)	-	-	-	89.7	142.7	302.4	c3
70	L12138_at	NM_019179.1	*Tyms*	thymidylate synthase (tsase)	8.5	6.8	53.4	-	-	-	c19
71	U89282_at	NM_022591.1	*Tep1*	telomerase protein component 1 (tlp1)	-92.4	-58.0	66.0	-	-	-	c19
**72**	**U14647_at**	**NM_012762.2**	***Casp1***	**caspase-1**	**56.8**	**282.8**	**442.9**	**53.0**	**253.6**	**479.3**	**c11**
**73**	**S79676_s_at**	**NM_012762.2**	***Casp1***	**caspase-1**	**-**	**-**	**-**	**153.5**	**361.4**	**508.0**	**c3**
74	J05122_at	NM_012515.1	*Bzrp*	peripheral-type benzodiazepine receptor	116.0	468.5	964.8	121.8	424.7	1241.2	c15
75	J02962_at	NM_031832.1	*Lgals3*	galectin-3	23.7	282.9	470.9	10.9	266.4	751.6	c11
76	L21711_s_at	NM_012976.1	*Lgals5*	galectin-5	175.3	1620.0	2164.8	240.5	1713.2	3658.9	c11
77	U72741_g_at	NM_010708.1	*Lgals9*	galectin-9	723.4	2339.9	2827.1	605.9	2388.0	4132.9	c11
78	U72741_at	NM_010708.1	*Lgals9*	galectin-9	90.4	447.0	388.1	74.3	400.3	566.9	c7
79	rc_AI104781_at	NM_017260.1	*Alox5ap*	5-lipoxygenase activating protein (FLAP)	45.53	116.35	176.86	35.42	81.00	131.91	c11
80	AB000199_at	NM_139329.1	*Cca2*	cca2	-10.36	18.14	101.83	-	-	-	c19
81	rc_AA998164_s_at	NM_171991.1	*Ccnb1*	cyclin b1	61.05	38.71	110.33	41.28	45.38	107.2	c14
82	D16308_at	XM_216276.1		cyclin d2	-	-	-	222.21	34.01	31.73	c20
83	AF036548_at	NM_054008.1	*Rgc32*	rgc-32	-	-	-	116.07	9.22	9.5	c20

**Growth control/neuroplasticity**											

84	A09811cds_s_at	NM_013122.1	*Igfbp2*	insulin-like growth factor binding protein 2 precursor	218.1	744.1	1292.1	-	-	-	c19
**85**	**X52498cds_at**	**NM_021578.1**	***Tgfb1***	**transforming growth factor beta 1 precursor **	**59.3**	**199.0**	**324.5**	**115.2**	**106.2**	**327.1**	**c15**
**86**	**AF089825_at**	**NM_031815.1**	***Inhbe***	**activin beta-e chain**	**146.2**	**151.4**	**40.9**	**-**	**-**	**-**	**c12**
87	S79820_at	NM_172563.1	*Hlf*	hepatic leukemia factor	-	-	-	207.0	88.9	16.3	c20
**88**	**E03082cds_s_at**	**NM_031073.1**	***Ntf3***	**neurotrophin-3 precursor (nt-3) **	**68.6**	**103.4**	**11.3**	**-**	**-**	**-**	**c12**
89	X62322_g_at	NM_017113.1	*Grn*	granulins precursor	543.09	1259.51	1686.5	544.37	1010.75	1952.91	c11
90	X06916_at	NM_012618.1	*S100a4*	nerve growth factor induced protein 42a	211.43	616.49	558.56	133.32	566.98	1346.52	c10

**Neurotransmission**											

91	AF020757_s_at	NM_053656.2	*P2rx2*	p2x purinoreceptor 2	-	-	-	54.41	28.99	20.25	c20
92	D63665_at	NM_057124.1	*P2ry6*	p2y purinoreceptor 6	84.30	312.05	447.53	44.77	213.71	493.88	c11
93	D00833_g_at	NM_013133.1	*Glra1*	glycine receptor alpha-1 precursor	-	-	-	57.99	42.25	16.72	c20

**Ionic homeostasis**											

94	U75916_at	NM_011597.1	*Tjp2*	zonula occludens 2	-	-	-	236.4	633.7	576.1	c2

**Lipid metabolism**											

95	AA850219_at	NM_012823.1	*Anx3*	lipocortin-iii	-	-	-	30.8	61.5	101.9	c3

**Miscellaneous**											

96	M86389cds_s_at	NM_031970.1	*Hspb1*	heat shock 27 kda protein (hsp27)	-264.3	112.6	72.1	-157.6	75.5	173	c7
97	U13177_at	NM_031237.1	*Ube2d3*	ubiquitin-conjugating enzyme E2D 3	414.3	220.7	87.2	-	-	-	c12
98	D10729_s_at	NM_080767.1	*Psmb8*	proteasome subunit beta type 8 precursor	51.9	216.6	449.7	74.1	246.4	579.4	c15
99	D10757_at	NM_012708.1	*Psmb9*	proteasome subunit beta type 9 precursor	30	35.3	96.6	4	76.4	131.9	c15
100	D10757_g_at	NM_012708.1	*Psmb9*	proteasome subunit beta type 9 precursor	148.7	820.5	1059.9	120	806.5	1644.2	c11
101	D45249_g_at	NM_017264.1	*Psme1*	proteasome activator complex subunit 1	-	-	-	2145.6	4508.6	7335.3	c3
102	D45250_s_at	NM_017257.1	*Psme2*	proteasome activator complex subunit 2	405.4	988.1	1494.7	439.3	1047.9	1876	c11
