# Supplementary material for: Gene expression in cortex and hippocampus during acute pneumococcal meningitis
Source: BMC Biol. 2006 Jun 2;4:15. doi: 10.1186/1741-7007-4-15 (PMC1523193; doi:10.1186/1741-7007-4-15)
Supplement: Additional file 1 — Implementation of the literature profiling algorithm by Chaussabel and Sher. [file 1741-7007-4-15-S1.doc]

**Our implementation of the literature profiling algorithm of Chaussabel and Sher**

We implemented the literature-profiling algorithm of Chaussabel and Sher [1] in a Perl program. The program writes and executes the search query for all gene names and their aliases using the syntax published by the NCBI for automated searching of MEDLINE [2]. The program further determines, for each gene, the percentage of abstracts containing each word found in the vocabulary extracted from all abstracts downloaded. For our purposes, we defined a word as any string composed by at least 3 characters (numerical strings were discarded). The term-occurrence values of redundant singular/plural forms were averaged using the Damian Conway's Perl module Lingua::EN::Inflect version 1.88 [3]. To eliminate ubiquitous terms and select only those that can be found in most abstracts of gene-specific collections and show a low baseline occurrence in the literature, a set of filters were successively applied to the raw data. First, the baseline occurrence for each term was estimated by taking their average occurrence in abstracts retrieved for a set of 250 genes randomly picked from Unigene Rat section [4] . The cut-off baseline occurrence was empirically set at 2.5%, since with this value most ubiquitous terms (e.g. "what", and "cell") were eliminated. Terms of which the occurrence was higher than the cut-off baseline were eliminated from the vocabulary of the experimental set of genes. For the remaining vocabulary, the differential cut-off between occurrence of a gene term in the experimental set and its baseline occurrence was optimized by applying the following equation: *cut-off = t + (k/n)* where *t* is the minimum threshold, *k* is a constant and *n* is the number of abstracts retrieved for a given gene; we arbitrarily set *t* = 15% and *k* = 1.5. Once again, terms with occurrence above the optimized cut-off were eliminated to compensate the difference in the number of abstracts retrieved for each gene. Terms present in the vocabulary of more than 20% of the genes in the data set were discarded to improve gene-term specificity, and only terms found to pass the filters for at least two of the genes in the data set were further retained, since a term can only be useful for defining relationships among genes if it is shared by at least two of them. The output of our program can be described as a term-by-gene matrix of term-frequencies.

**References**
