# Supplementary figures and images for: Gene expression in cortex and hippocampus during acute pneumococcal meningitis
Source: BMC Biol. 2006 Jun 2;4:15. doi: 10.1186/1741-7007-4-15 (PMC1523193; doi:10.1186/1741-7007-4-15)

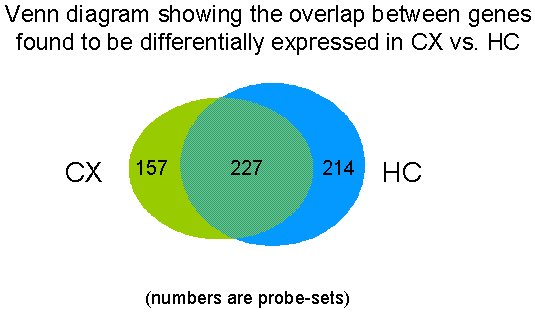

Supplement: Additional file 2 — Schematic of spatial overlap in gene expression between cortex and hippocampus. [file 1741-7007-4-15-S2.jpeg]
